# Assessing the acceptability and feasibility of remote spirometric monitoring for rural patients with interstitial lung disease: a multimethod approach

**DOI:** 10.1186/s12931-024-02735-z

**Published:** 2024-02-20

**Authors:** Ryan D. Boente, Sydney Schacht, Rebecca Borton, Joseph Vincent, Lilian Golzarri-Arroyo, Nicholas Rattray

**Affiliations:** 1 Department of Medicine, Division of Pulmonary, Critical Care, Allergy, Sleep and Occupational Medicine, Indiana University School of Medicine, 1120 W. Michigan St, Gatch Hall, CL 290B, Indianapolis, IN 46202 USA; 2 Roudebush VA Medical Center, Center for Health Information and Communication, Indianapolis, IN USA; 3 PatientMpower, Dublin, Ireland; 4 Department of Epidemiology and Biostatistics, Indiana University School of Public Health, Bloomington, IN USA; 5 Regenstrief Institute, Inc., Indianapolis, IN USA; 6 Department of Medicine, Indiana School of Medicine, Indianapolis, IN USA

**Keywords:** Interstitial lung disease, Remote patient monitoring, Rural, Multimethod, Patient experience

## Abstract

**Introduction:**

Interstitial lung disease encompasses a group of rare lung conditions causing inflammation and scarring of lung tissue. The typical method of monitoring disease activity is through pulmonary function tests performed in a hospital setting. However, accessing care can be difficult for rural patients due to numerous barriers. This study assesses the feasibility and acceptability of home spirometry telemonitoring using MIR-Spirometers and the patientMpower home-monitoring platform for rural patients with interstitial lung disease.

**Methods:**

Unblinded, uncontrolled, prospective, multiple-methods study of the feasibility and utility of remote monitoring of 20 rural subjects with interstitial lung disease. Study assessments include adherence to twice weekly spirometry for 3 months in addition to mMRC dyspnea and EQ-5D-5L health-related quality of life questionnaires with each spirometry maneuver. Upon completion, subjects were encouraged to complete an 11-question satisfaction survey and participate in semi-structured qualitative interviews to further explore expectations and perceptions of rural patients to telehealth and remote patient monitoring.

**Results:**

19 subjects completed the 3-month study period. Adherence to twice weekly spirometry was mean 53% ± 38%, with participants on average performing 2.26 ± 1.69 maneuvers per week. The median (Range) number of maneuvers per week was 2.0 (0.0, 7.0). The majority of participants responded favorably to the patient satisfaction survey questions. Themes regarding barriers to access included: lack of local specialty care, distance to center with expertise, and time, distance, and high cost associated with travel. Remote monitoring was well perceived amongst subjects as a way to improve access and overcome barriers.

**Conclusions:**

Remote spirometry monitoring through web-based telehealth is acceptable and feasible for rural patients. Perceived benefits include overcoming access barriers like time, distance, and travel costs. However, cost, reimbursement, and internet access must be addressed before implementing it widely. Future studies are needed to ensure long-term feasibility and to compare outcomes with usual care.

**Supplementary Information:**

The online version contains supplementary material available at 10.1186/s12931-024-02735-z.

## Introduction

Interstitial lung disease (ILD) describes a group of rare lung conditions characterized by inflammation and scarring of lung tissue [1]. The most common form, idiopathic pulmonary fibrosis (IPF), typically progresses slowly over time and is associated with a significant reduction in quality of life as the disease progresses [2–4]. While IPF is the most common, dozens of other etiologies of ILD exist, and the natural course of ILD varies depending on the variety and numerous other factors. The typical model for monitoring disease activity is through the use of in-hospital pulmonary function tests (PFTs) to measure forced vital capacity (FVC) of the lungs [5]. Patients need frequent contact with their medical team to perform PFTs to determine the appropriateness of therapeutic options. Given the complexities, patients seek care at expert centers with experience caring for individuals with ILD.

Rural patients with ILD face unique barriers affecting their access to subspecialty care [6, 7]. Patients living in a rural setting are significantly impacted by distance, time, and cost regarding travel to physicians and clinics with expertise [6–8]. Health disparities for rural patient populations are well known and result in significantly worse health outcomes, as evidenced by age-adjusted death rates being nearly 20% higher for rural versus urban individuals, according to 2021 National Center for Health Statistics data [9]. There is a pressing need to identify cost-effective, patient-friendly ways to improve access to care for those living with ILD.

Telehealth creates a unique opportunity to enhance access to expert care [10, 11]. Home monitoring allows for care at a distance, potentially overcoming some geographical barriers that impact rural patients who often live in remote areas. One limitation of previous telehealth solutions was the inability to monitor disease activity for patients with ILD. Utilizing a home-based spirometry platform within a telehealth program may allow for closer monitoring without compromising care. Studies evaluating feasibility, reliability, and adherence to home spirometry in ILD exist in several contexts, with variable results reported. Adherence to home spirometry in general cohorts of ILD and IPF subjects ranges from 25 to 98.8% and adherence typically wanes with time [10, 12, 13]. Studies with comprehensive home monitoring programs that had built-in infrastructure and support to enroll, monitor, and remind subjects to perform spirometry had the highest levels of adherence [14]. Studies have shown that home spirometry measurements highly correlate with in-hospital measurements of FVC (r = 0.94; p < 0.001), but with slightly lower readings at home [13]. However, no such studies have been conducted in rural patient populations. Studies are needed to determine the acceptability and feasibility of a remote spirometry platform in combination with telehealth as a modality to improve access to care of ILD in a real-world clinical care setting, particularly in rural patient populations who may or may not be as receptive to technology-based solutions.

In this study, we aim to assess the feasibility of home spirometry telemonitoring using Bluetooth-enabled MIR-Spirometers and a web-based platform to monitor lung function in rural subjects with ILD by assessing adherence to performing twice weekly home spirometry. Secondary aims are to assess the attitudes and experiences of rural subjects to remote patient monitoring while identifying barriers and facilitators to its use in clinical care using a multiple-methods approach. We hypothesize that most rural patients with ILD will find remote monitoring feasible and acceptable; however, there will be differences in adherence and acceptability among participants.

## Methods

### Design and setting

This is a single-arm, prospective, multiple-methods study of the feasibility and utility of remote monitoring via the patientMpower platform for the management of rural participants with ILD when used in conjunction with usual care. PatientMpower is a Dublin, Ireland-based digital healthcare company with affiliate sites in the United States. It provides virtual care solutions for people with chronic conditions, including ILD. This study was performed at Indiana University (IU), a large tertiary medical and transplant center in Indianapolis, Indiana, with Indiana University Institutional Review Board approval (IRB approval #16165) and in accordance with the Declaration of Helsinki. Convenience sampling was used to recruit participants already receiving care at the IU ILD clinic. Participants were screened using the electronic medical record and were considered if they resided in a region classified as rural and had confirmed ILD. Following informed, written consent twenty (20) participants meeting inclusion criteria were enrolled and followed for 3 months following the first use of their home spirometer device with a web-based platform assessing adherence to performing twice weekly spirometry. We used a variety of methods to assess the overall attitudes towards telehealth and the satisfaction of participants with a home-based monitoring platform that required a web application. We conducted written patient satisfaction surveys and offered an optional semi-structured interview to gain a better understanding of the barriers and facilitators to remote monitoring of rural participants with ILD. Sample size and duration were based on evaluating feasibility.

### Study procedures

Screening was initiated by reviewing consecutive patients evaluated at the IU ILD clinic starting January 1, 2022. Patients identified as living in a region designated as “Rural” based on the 2013 National Center for Health Statistics Urban–Rural Scheme using patient-reported zip codes and having an ILD diagnosis as defined by ICD-10 codes (J84 and subset) were contacted by phone to discuss the study. Patients who were pregnant or did not have access to the internet, smartphone, tablet, or computer with internet capabilities were excluded. Written consent was obtained in the clinic or by mail.

Study participants received education and training on how to download the web application to their device (smartphone or tablet), pair it with Bluetooth-enabled SpiroBank SMART spirometers, and interface with the device in person or virtually. Additional educational materials about the platform and spirometry were provided by patientMpower with the spirometer. Participants were considered appropriately trained after producing three reproducible FVC measurements with less than 150 ml variation among them.

During the study, the participants were instructed to use the platform and perform spirometry at least twice per week. They were also required to answer patient-reported outcome questionnaires that were integrated into the web application with each spirometry maneuver. The participants were followed for three months.

Upon completion of the study, participants were asked to complete a written patient satisfaction survey and given an opportunity to be interviewed to share their experiences with using the patientMpower platform and home spirometry to monitor their lung disease.

### Data collection

At inclusion, we retrospectively collected demographic and clinical data from the electronic medical record, including age, sex, race, height, ILD phenotype, and PFT data: FVC and Forced Expiratory Volume in one second (FEV1) (Table 1).

**Table 1:** Patient characteristics

Characteristic	N = 19
Age (at enrollment)	
Mean (SD)	55 (15)
Median (Range)	57 (24, 75)
Sex	
F	11 (58%)
M	8 (42%)
Race	
Black	1 (5.3%)
White	18 (95%)
FVC (prior to start)	
Mean (SD)	3.04 (0.83)
Median (Range)	3.09 (1.57, 4.75)
Unknown	2
FEV1 (prior to start)	
Mean (SD)	2.38 (0.53)
Median (Range)	2.36 (1.45, 3.48)
Unknown	1
ILD Type	
Chronic Hypersensitivity Pneumonitis	1 (5.3%)
CPFE	2 (11%)
CTD-ILD	3 (16%)
Idiopathic NSIP	1 (5.3%)
ILD	1 (5.3%)
IPF	4 (21%)
LAM	2 (11%)
NSIP	1 (5.3%)
Sarcoidosis	2 (11%)
Unclassifiable	2 (11%)

n (%)

*CPFE* Combined Pulmonary Fibrosis and Emphysema

*CTD−ILD* Connective Tissue Disease−related Interstitial Lung Disease

*FEV1* Forced Expiratory Volume in 1 s

*FVC* Forced Vital Capacity

*ILD* Interstitial Lung Disease

*IPF* Idiopathic Pulmonary Fibrosis

*LAM* Lymphangioleiomyomatosis

*NSIP* Non−Specific Interstitial Pneumonia

During the 3-month follow-up period, participants were asked to perform home spirometry maneuvers and answer patient-reported questionnaires twice weekly. The results from forced spirometry measurements (FVC and FEV1) performed at home and responses to patient-reported outcome measures (PROM) for the health-related quality of life were automatically recorded in the patient-facing application at each use and securely transmitted to the investigator portal in real-time. Participants were asked to complete (EQ-5D-5L) and the Modified Medical Research Council (mMRC) breathlessness scales [15, 16]. All data (spirometry and PROM) collected during the 3-month study period were considered for each participant. Home spirometry readings outside the 1st and 99th percentile of the aggregated group data were excluded as outliers and considered substandard blows. Participation in the study was discontinued only if the participant withdrew consent and data up to the point of withdrawal was included.

### Patient satisfaction survey

An 11-question patient satisfaction survey was developed based on a literature review on telehealth and home spirometry [17–20]. Surveys were used to assess rural participants’ attitudes and experiences by asking general questions regarding comfort, feasibility, and acceptability, with responses scored on a 1–5 Likert scale, with 1 being strongly disagree and 5 being strongly agree. Surveys were open for completion for up to 3 months following completion of the study, and results were anonymous. 11 of 19 participants completed surveys.

### Interviews

We then conducted semi-structured interviews to explore further the experiences and perceptions of rural subjects to home monitoring. The 13-question interview guide was developed based on a literature review [10]. Participants were recruited by phone and email, and an independent study coordinator conducted interviews. Post-interview observations and analytical memos were completed by (RDB, RB, SS, and NR). Interviews ranged from 20 to 40 min, were audio recorded, and transcribed verbatim using TranscribeMe transcription services [21]. A total of 11 participants were interviewed.

### Analyses

#### Statistical analysis

Adherence was measured as the number of days the patient provided a reading or interacted with the platform divided by the total number of study days. Descriptive statistics were used to evaluate the PROMs (EQ-5D-5L and mMRC). Weekly adherence to spirometry was measured as the number of weeks the patient provided two spirometry readings divided by the total number of study weeks. Acceptability will be assessed using the patient satisfaction survey completed at the conclusion of the study.

#### Qualitative analysis

Analysis of interviews incorporated deductive categories derived from the interview guide, and inductive themes emerged from the interviews. RDB, RB, SS, and NR analyzed the transcripts by developing a qualitative codebook and applying codes to interview excerpts through an iterative, consensus-based approach. The team then compared the provisional codes, and differences were reconciled to build an inventory of codes. Themes from the survey were used deductively as a set of codes along with the codes that emerged from the open coding, and the resulting codebook was applied to each of the transcripts [22, 23]. Discrepancies were resolved in bi-monthly meetings, and data and coding schemes were revised until thematic saturation was achieved [24].

## Results

A total of 20 participants from the IU ILD clinic meeting inclusion criteria were consented, with one dropping out before study interventions due to personal reasons for a final study cohort of 19. Baseline characteristics are included in (Table 1). Most participants were white (95%) and female (58%), with a median age of 57 (24, 75) and encompassing a wide range of ILD subtypes (Additional file 1).

### Adherence

**Fig. 1:**
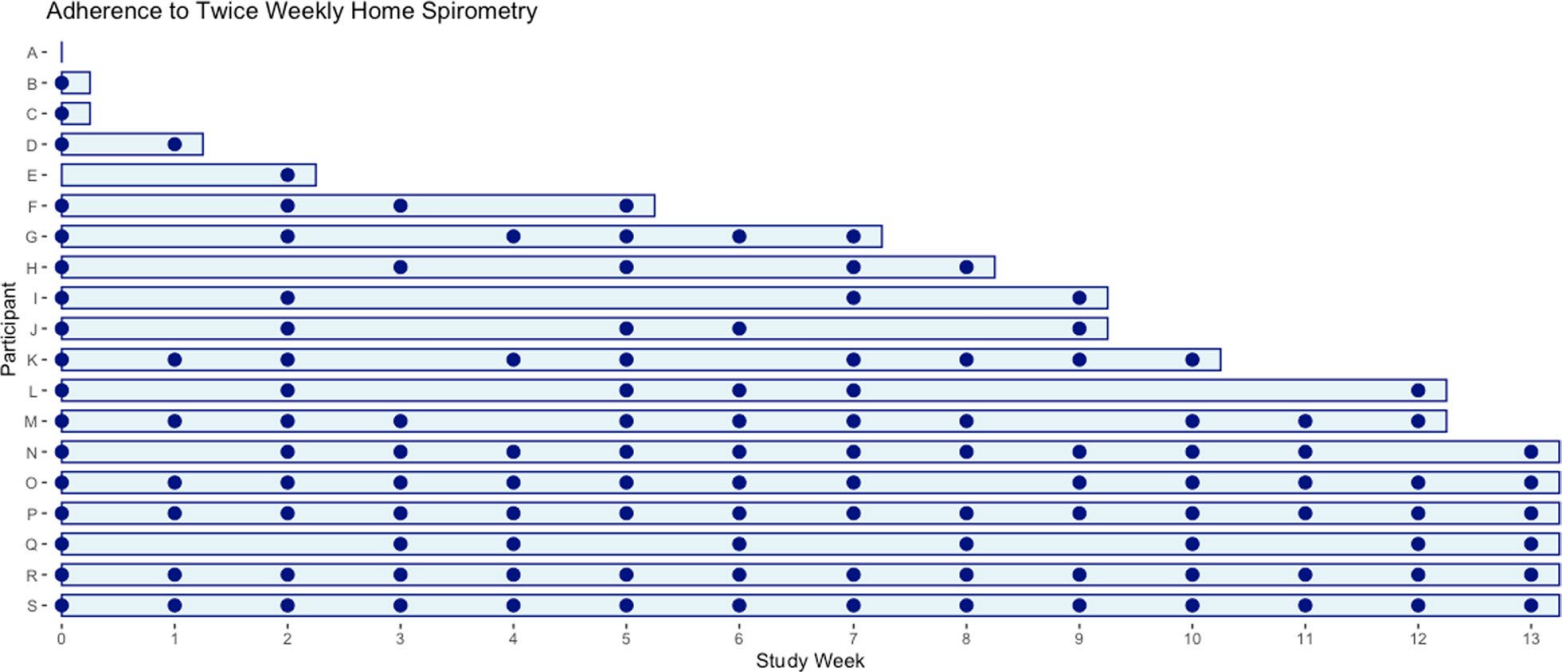
Participant adherence to twice-weekly spirometry. This graph shows the adherence of individual participants to the twice-weekly spirometry over the course of the study. The X-axis labels each participant as A-S, while the Y-axis shows the study week. Each dot on the graph represents the adherence of an individual participant to the spirometry for the respective study week

Adherence to twice weekly spirometry was mean 53 ± 38%, with participants on average performing 2.26 ± 1.69 maneuvers per week with additional patient recorded outcome measures including dyspnea (mMRC) and healthcare-related quality of life (EQ-5D-5L) values reported (Table 2). The adherence of individual participants to performing twice-weekly spirometry varied greatly, as shown in Fig. 1**.** We conducted a post hoc analysis and found adherence to once-weekly spirometry was much higher at 68%.

**Table 2:** Adherence data

Characteristic	N = 19
2x/week spirometry adherence	
Mean (SD)	0.53 (0.38)
Median (Range)	0.46 (0.00, 1.00)
Average # maneuvers/week	
Mean (SD)	2.26 (1.69)
Median (Range)	2.00 (0.00, 7.00)
mMRC Average	
Mean (SD)	1.19 (1.00)
Median (Range)	1.14 (0.00, 2.67)
Unknown	10
EQ-5D-5L Average	
Mean (SD)	0.83 (0.14)
Median (Range)	0.81 (0.54, 1.00)
Unknown	4

### Attitudes and experiences

#### Patient satisfaction surveys

**Fig. 2:**
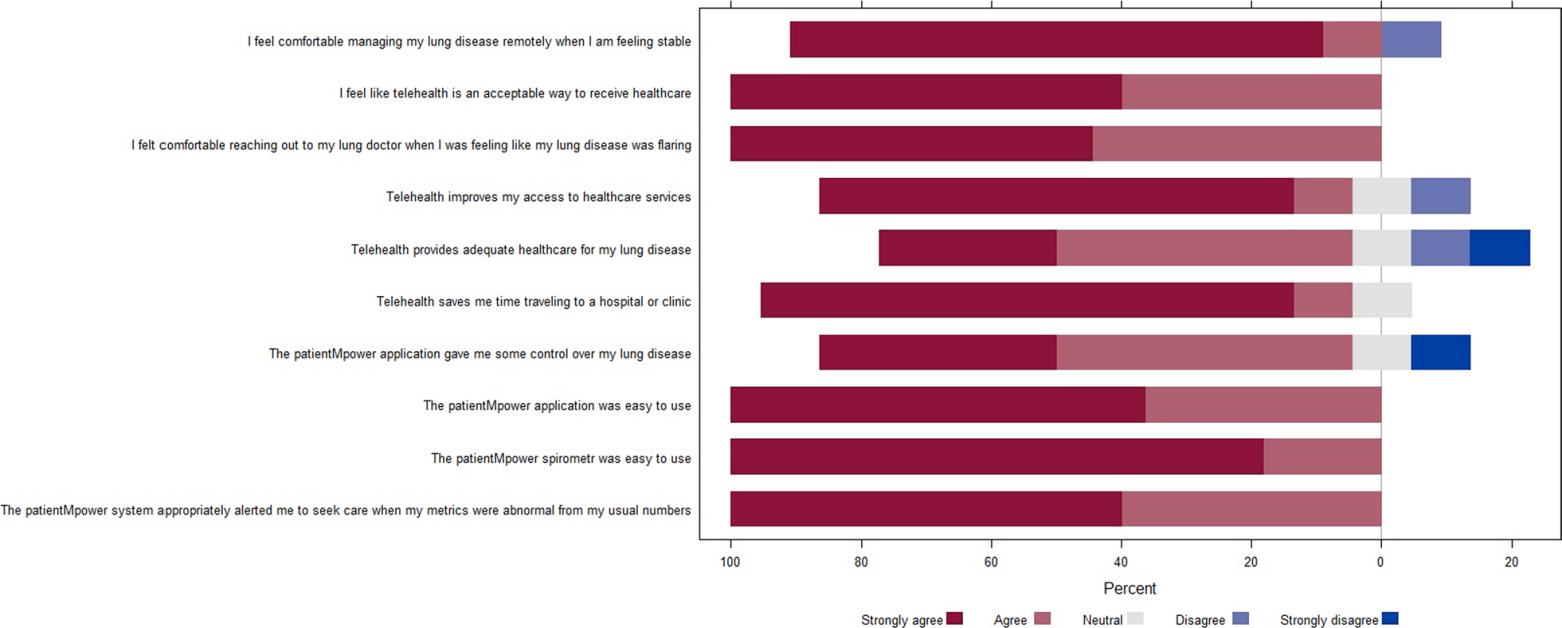
Satisfaction survey responses. This is a Likert plot that displays the responses of participants to the satisfaction survey questionnaire. The Y-axis represents individual survey questions and responses are color-coded based on the range of responses from strongly agree to strongly disagree. The bars represent the percentage of individuals for each respective response

11 out of 19 participants completed anonymous patient satisfaction surveys. The surveys showed overwhelmingly positive responses to questions regarding telehealth and remote spirometry monitoring to manage their lung disease (Fig. 2). For example, the majority either strongly agree or agree that telehealth utilizing a web-based platform improves access and is an acceptable and adequate way to receive healthcare while giving individuals the sense of having some control of their lung disease. All subjects felt that the spirometer and patientMpower web applications were easy to use. One survey question concerning whether participants would continue to use the patientMpower application and spirometer in the future was censored from the analysis as the items on the Likert scale for that question differed from that of the other ten questions (Additional file 2).

#### Qualitative interviews

11 of 19 participants agreed to the optional qualitative interviews, which lasted between 8 and 31 min. Themes, subthemes, and exemplar quotes are illustrated in Table 3.

**Table 3:** Qualitative Interviews

Barriers to ILD care		
Themes	Categories	Examples:
Travel	Distance	“I think the biggest challenge is just the distance that you have to travel from home to Indy. It's a three-hour drive for us”
	Travel burden	“I had to bring my own oxygen. So I think I had 32 oxygen tanks in the back of her truck”
Cost	Money	“The money, the cost to drive to Indy can be a burden” “I mean, they're (PFTs) expensive”
	Time	“it's a 90-min drive each way just to go to the doctor's office”
(Un)Availability of local expertise	Healthcare	“A small town might have its advantages, but when it comes to healthcare, not so much”
	ILD familiarity	“In my area, people don't really know about it”
	Resources	“They don't have the equipment (PFTs)”

Facilitators to ild care: telemedicine/home monitoring		
Themes	Categories	Examples:
Improved Access	Health care team	“I think you might be able to get in sooner”
	Resources	“You can do it at home (PFTs)”
Savings	Time	“And you don't have to travel down to Indy with no oxygen tank.”
	Money	“It's something that's probably very cost-effective for both patients and doctor”
Home Monitoring	Awareness	“While I don't know what some of the numbers mean, I can certainly see the trends, and if I see the trends going badly, then it gives me an opportunity to call the doctor's office”
	Empowerment	“I do believe it empowers the patient to be more of an advocate for their own care”

Technology: merits/limitations	
Themes	Examples
Merits	“Well, I think technology is the way of the world now, so I think the right devices and the right things to help manage your disease, it can't do nothing but help” “Well, it's better, and it's faster, and it's much more convenient”
Limitations	“One disadvantage is they can't actually see you hands on. He can't listen to my lungs on telehealth or something like that” “I just think personal contact is important” “The disadvantages are, as I mentioned, the low signal strength and just, essentially, lack of coverage where I live”

*ILD* interstitial lung disease, *PFT* pulmonary function test

The table depicts the themes which we developed from analyzing data about barriers to ILD care in general, and how telemedicine and remote monitoring with spirometry can serve as a facilitator to improved access and overcome common barriers to ILD care for patients in rural areas

#### Theme 1: barriers to care

Most participants cited travel distance as one of the primary barriers to receiving care for their ILD. For example, one of many similar quotes highlights this burden, “I think the biggest challenge is just the distance that you have to travel from home to Indy [Indianapolis]. It's a three-hour drive for us”.

For many, the cost of travel was compounded by additional burdens, such as traveling with oxygen. The cost of travel often extended beyond gas prices to that of family and friends missing time from work to accompany participants to clinic appointments (Additional file 3).

The reason for pursuing ILD care despite these burdens was due to the unavailability of local expertise and resources; as one participant explained, “A small town might have its advantages, but when it comes to healthcare, not so much”.

#### Theme 2: facilitators to care

Telemedicine and home monitoring were consistently viewed as facilitators of ILD care. Patients suggested that telemedicine not only could improve access but also may be efficient: “it’s something that’s probably cost-effective for both patients and doctor.” Furthermore, participants felt more aware of their lung disease and that home monitoring can empower patients to advocate for their own care.

#### Theme 3: merits and limitations of technology

Technology was viewed positively, for example, “Well, it's better, and it's faster, and it's much more convenient”. Disadvantages included the lack of human touch and one-on-one connection that occurs with in-person visits. Additionally, one participant noted limitations due to poor broadband internet access where he resides.

## Discussion

This is the first study evaluating the acceptability and feasibility of home monitoring in an ILD cohort of rural patients, a population with known barriers to accessing care that could be addressed by home monitoring programs. The aim was to examine unique challenges and opportunities in implementing home monitoring for ILD in rural patient populations.

Using a multi-method approach, we found that nearly all surveyed participants offered favorable perspectives on the acceptability of home monitoring to manage their lung disease. We distilled themes from qualitative interviews that highlight common challenges including lack of locally available care as well as various travel burdens that limit access. Telehealth utilizing a remote monitoring platform was viewed as an effective way to overcome barriers and improve access to care while simultaneously giving individuals more insight into their lung disease and a sense of empowerment in disease self-management and awareness. These findings suggest that interest in remote monitoring among rural ILD patients is considerable.

Despite overwhelmingly positive feedback from the acceptability surveys and qualitative interviews, adherence to twice-weekly spirometry was modest at 53% for our rural cohort, whereas many other studies report patient adherence in the range of 70–90% [25, 26]. Adherence numbers are partially skewed because of significant variability among participants. For example, 7 of 19 participants adhered to twice-weekly spirometry less than one-third of the time, of which three were only adherent one week or less out of the 13-week study period. Nevertheless, the adherence data suggests that barriers to use may exist, which may be specific to rural patient populations and/or due to technical issues with the implementation and monitoring of subjects during the study period.

Lower adherence than expected may be due to numerous factors. First, limited infrastructure was available to train and monitor participants throughout our study. Due to the scope, budget, and timeline of our project, many participants were recruited and thoroughly trained initially on the use of spirometers and platforms, but we had to rely on periodic push notification reminders to perform spirometry. This contrasts with a trial out of the Netherlands that achieved very high adherence (Median 97%, Mean 93%) to daily spirometry through a more comprehensive home monitoring program with more intensive onboarding and layered levels of alerts to participants and study team members when spirometry was not being performed [14]. These differences between our study and theirs highlight how infrastructure and support are facilitators to implementing a remote monitoring platform.

While the optimal frequency for home spirometry measurements is unknown, studies performing daily spirometry had much higher levels of adherence [10]. Furthermore, other studies have shown that daily spirometry allows for quicker evaluation of clinical deterioration and that daily spirometry can detect variability in FVC, which is linked to disease progression in ILD [27, 28]. We aimed to balance frequency with patient burden by choosing a target of twice weekly. However, it is possible that it may be more challenging for patients to remember to do spirometry twice weekly than other frequencies. Daily spirometry was reported as not burdensome by 90% of IPF participants in one study and, therefore may be a more reasonable approach to achieving higher patient adherence [13]. Ultimately, decisions on monitoring frequency for future studies and home monitoring programs will largely depend on the scope and goals of individual programs, but this study adds to the literature that suggests that ILD patients can feasibly and reliably perform home spirometry.

Lastly, it is likely that a subgroup of rural patients may have additional barriers to home monitoring using a web-based platform. One barrier identified was difficulties with reliable internet access, further widening health disparities for this individual, and considerations for additional accommodations such as devices with cellular capabilities or other methods to participate in home monitoring should be considered.

While the scope of our study was acceptability and feasibility, there are several limitations to mention. The satisfaction surveys and qualitative interviews were optional and anonymous, which prevented linking these results to adherence data. It is possible that those with poor adherence had significant limitations and barriers not captured in the qualitative interviews and had lower satisfaction with the monitoring platform. Studies are needed to understand better the reasons for low adherence in subsets of patients to investigate how to address additional barriers to avoid exacerbating health disparities for select rural patients with ILD.

When implemented according to protocol and with significant support, home monitoring programs can lead to high adherence, generating home-spirometry values that reliably correlate with hospital-performed spirometry [14]. Infrastructure and support are crucial for the success of home monitoring programs, highlighting the need for funding mechanisms for sustainability before widespread implementation. Centers for Medicare and Medicaid Services (CMS) will reimburse remote patient monitoring for set-up, remote monitoring, interactive communication, and interpretation of data, with some codes eligible for monthly reimbursement. However, reimbursement requirements are stringent, typically requiring that a patient record measurement from a connected device for at least 16 out of 30 days in a calendar month (to be eligible for reimbursement).

Based on the results of our study and a review of the literature, we propose the following as a research design to further assess the feasibility and utility of remote patient monitoring for rural patients with ILD in a way that is reimbursable by CMS and overcomes some of the challenges and limitations we encountered during our study. Participants will perform daily spirometry to increase the likelihood of generating 16 days of data, fulfilling criteria for reimbursement, as the sustainability of remote monitoring programs will depend on ongoing funding and support. Detailed, in-person training of subjects and family members on how to pair the spirometer with a phone or tablet and ensure they know how to interact with the web-based platform and perform spirometry maneuvers that correlate with hospital-performed values. Participants should be able to generate three reproducible FVC measurements, with < 150 ml difference in the highest FVCs and < 10% difference with hospital-performed measurements. Individuals without or limited access to Wi-Fi or broadband internet should be offered devices with cellular capabilities to ensure disparities are not widened for this subset of rural individuals with ILD. Layers of monitoring include push notifications by email and text to subjects after two consecutive days of not performing spirometry and an email alert to study or clinical team members after the third day without an FVC result or a decline in FVC by more than 10% on three consecutive days [14]. Monitoring platforms should have technical support mechanisms in place to troubleshoot challenges that arise.

## Conclusions

This study highlights the interest in remote patient monitoring among rural ILD patients. Although promising, significant challenges exist in implementing, monitoring, and sustaining such a program. Future research is necessary to ensure long-term feasibility and compare outcomes to usual care.

### Supplementary Information

**Additional file 1. **Patient satisfaction survey questionnaire. A patient satisfaction survey questionnaire was distributed to all participants at the study's conclusion. Results were reported on a Likert scale from 1 to 5.

**Additional file 2.** Adherence to once-weekly spirometry. Table depicting the results of a posthoc analysis of adherence to participant performing once-weekly spirometry.

**Additional file 3.** Individual participant adherence to one-weekly spirometry. Figure depicting individual participant adherence to weekly spirometry during the study period. Each dot represents a study participant's (A-S) adherence to weekly spirometry with respect to study week (0–13).

## Data Availability

The datasets generated and/or analyzed during the current study are available from the corresponding author on reasonable request.

## Abbreviations

CMS: Centers for Medicare and Medicaid services
CPFE: Combined pulmonary fibrosis and emphysema
CTD-ILD: Connective tissue disease-related interstitial lung disease
FEV1: Forced expiratory volume in one second
FVC: Forced vital capacity
ILD: Interstitial lung disease
IPF: Idiopathic pulmonary fibrosis
IU: Indiana University
LAM: Lymphagnioleiomyomatosis
mMRC: Modified Medical Research Council
NSIP: Nonspecific Interstitial Pneumonia
PFT: Pulmonary function test(s)
PROM: Patient reported outcome measure(s)
