# Supplementary material for: Assessing the acceptability and feasibility of remote spirometric monitoring for rural patients with interstitial lung disease: a multimethod approach
Source: Respir Res. 2024 Feb 20;25:92. doi: 10.1186/s12931-024-02735-z (PMC10877761; doi:10.1186/s12931-024-02735-z)
Supplement: Supplementary file 1 — Additional file 1. Patient satisfaction survey questionnaire. A patient satisfaction survey questionnaire was distributed to all participants at the study's conclusion. Results were reported on a Likert scale from 1 to 5. [file 12931_2024_2735_MOESM1_ESM.docx]

**Rural ILD Remote Monitoring Patient Satisfaction Questionnaire**

On a scale of 1-5, with 1 being strongly disagree and 5 being strongly agree, please answer the following questions:

Telehealth improves my access to healthcare services:

Strongly Disagree Disagree Neither Agree Strongly Agree

1 2 3 4 5 N/A

Telehealth saves me time traveling to a hospital or clinic:

Strongly Disagree Disagree Neither Agree Strongly Agree

1 2 3 4 5 N/A

Telehealth provides adequate healthcare for my lung disease:

Strongly Disagree Disagree Neither Agree Strongly Agree

1 2 3 4 5 N/A

The patientMpower application was easy to use:

Strongly Disagree Disagree Neither Agree Strongly Agree

1 2 3 4 5 N/A

The patientMpower application gave me some control over my lung disease:

Strongly Disagree Disagree Neither Agree Strongly Agree

1 2 3 4 5 N/A

The patientMpower spirometer was easy to use:

Strongly Disagree Disagree Neither Agree Strongly Agree

1 2 3 4 5 N/A

I feel comfortable managing my lung disease remotely when I am feeling stable:

Strongly Disagree Disagree Neither Agree Strongly Agree

1 2 3 4 5 N/A

I felt comfortable reaching out to my lung doctor when I was feeling like my lung disease was flaring:

Strongly Disagree Disagree Neither Agree Strongly Agree

1 2 3 4 5 N/A

The patientMpower system appropriately alerted me to seek care when my metrics were abnormal from my usual numbers:

Strongly Disagree Disagree Neither Agree Strongly Agree

1 2 3 4 5 N/A

I feel like telehealth is an acceptable way to receive healthcare:

Strongly Disagree Disagree Neither Agree Strongly Agree

1 2 3 4 5 N/A

If given the opportunity, how likely are you to continue to use the patientMpower application and spirometer in the future?

Very Unlikely Unlikely Neither Likely Very likely

1 2 3 4 5 N/A

Please provide general comments about your experience with home spirometry and the patientMpower application.

____________________________________________________________________________________________________________________________________________________________________________________________________________________________________________________________________________________________________________________________________________________________________________________________________________________________________________________________________________________________________________________________________________________________________________________________________________________________________________________________________________________________________________________________________________________________________________________________________________________________________________________________________________________________________________________________________________________________________________________________________________________________________________________________________________________________________________________________________________________________________________________________________________________________________________________________________________________________________________________________________________________________________________________________________________________________________________________________________________________________________________________________________________________________________________________________________________________________________________________________________________________
