# Supplementary figures and images for: Assessing the acceptability and feasibility of remote spirometric monitoring for rural patients with interstitial lung disease: a multimethod approach
Source: Respir Res. 2024 Feb 20;25:92. doi: 10.1186/s12931-024-02735-z (PMC10877761; doi:10.1186/s12931-024-02735-z)

## Slide 1
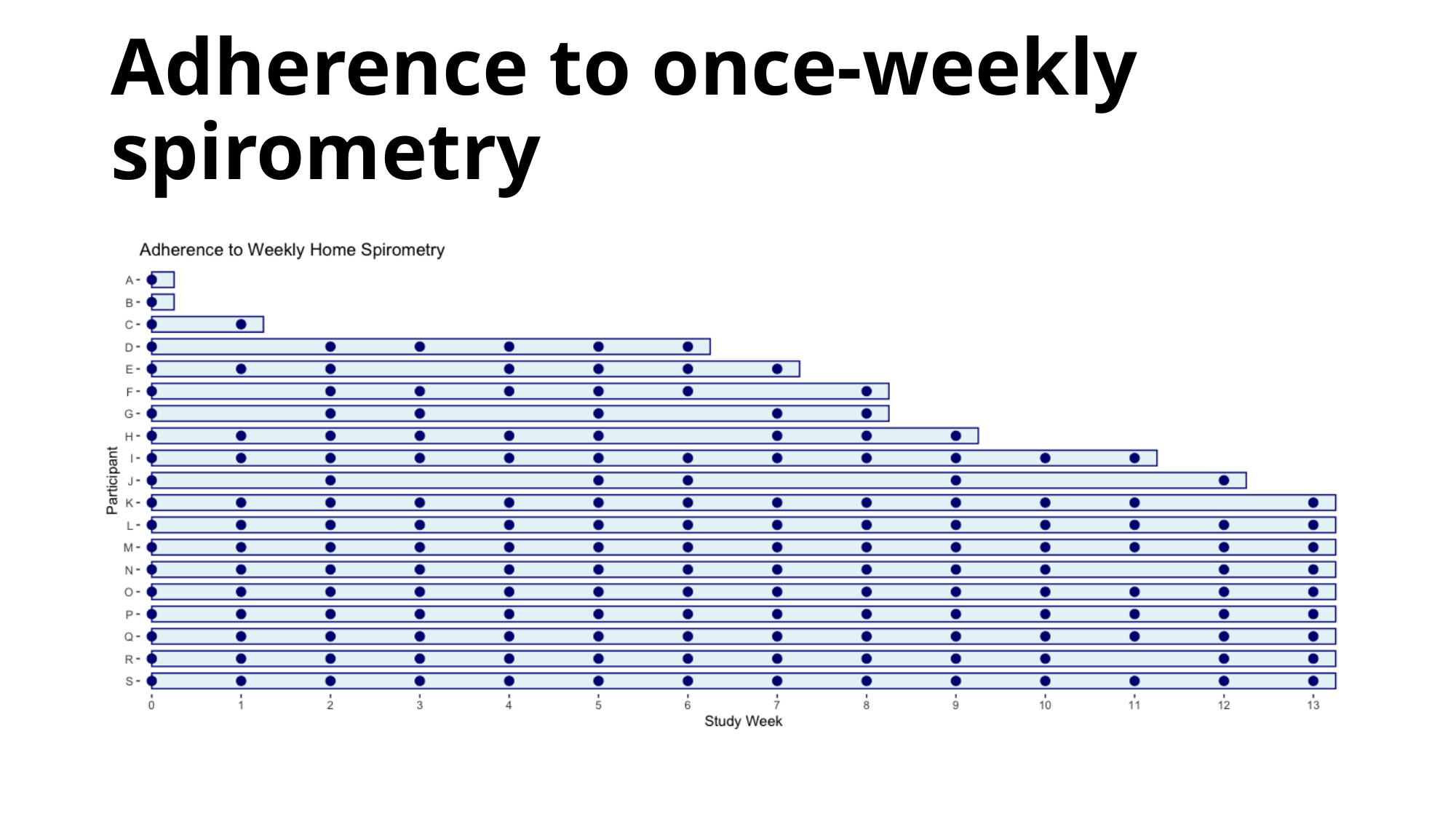

Adherence to once-weekly spirometry

Supplement: Supplementary file 2 — Additional file 2. Adherence to once-weekly spirometry. Table depicting the results of a posthoc analysis of adherence to participant performing once-weekly spirometry. [file 12931_2024_2735_MOESM2_ESM.pptx]

## Slide 1
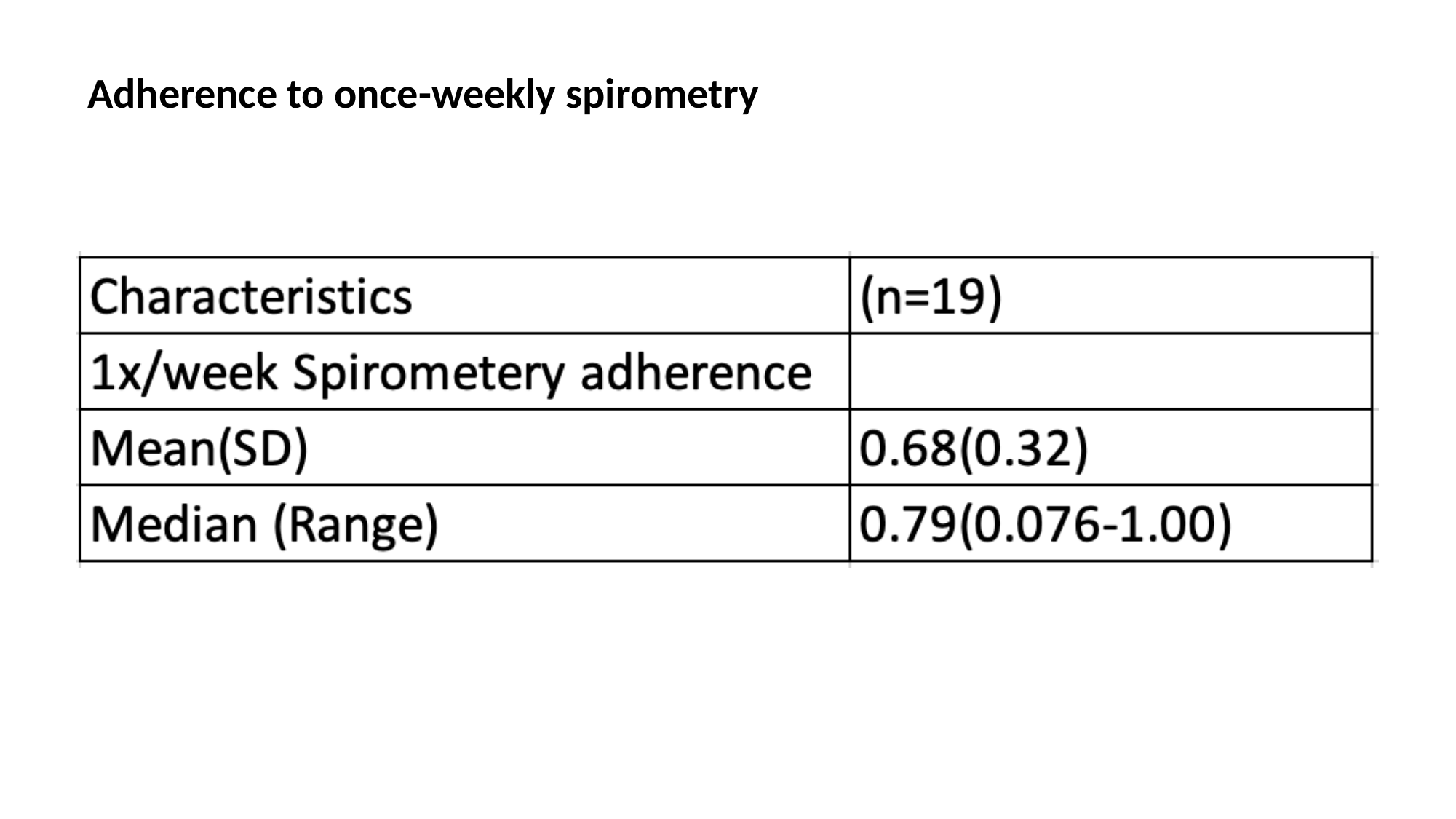

Adherence to once-weekly spirometry

Supplement: Supplementary file 3 — Additional file 3. Individual participant adherence to one-weekly spirometry. Figure depicting individual participant adherence to weekly spirometry during the study period. Each dot represents a study participant's (A-S) adherence to weekly spirometry with respect to study week (0–13). [file 12931_2024_2735_MOESM3_ESM.pptx]
